# Effects of endotoxin on lactate metabolism in humans

**DOI:** 10.1186/cc11444

**Published:** 2012-07-27

**Authors:** Burkhard Michaeli, Alexandre Martinez, Jean-Pierre Revelly, Marie-Christine Cayeux, René L Chioléro, Luc Tappy, Mette M Berger

**Affiliations:** 1 Service of Adult Intensive Care, Lausanne University Hospital (CHUV), Rue du Bugnon 46, Lausanne 1011, Switzerland; 2 Department of Physiology, University of Lausanne, School of Biology and Medicine, Rue du Bugnon 7, Lausanne 1011, Switzerland

## Abstract

**Introduction:**

Hyperlactatemia represents one prominent component of the metabolic response to sepsis. In critically ill patients, hyperlactatemia is related to the severity of the underlying condition. Both an increased production and a decreased utilization and clearance might be involved in this process, but their relative contribution remains unknown. The present study aimed at assessing systemic and muscle lactate production and systemic lactate clearance in healthy human volunteers, using intravenous endotoxin (LPS) challenge.

**Methods:**

Fourteen healthy male volunteers were enrolled in 2 consecutive studies (n = 6 in trial 1 and n = 8 in trial 2). Each subject took part in one of two investigation days (LPS-day with endotoxin injection and placebo-day with saline injection) separated by one week at least and in a random order. In trial 1, their muscle lactate metabolism was monitored using microdialysis. In trial 2, their systemic lactate metabolism was monitored by means of a constant infusion of exogenous lactate. Energy metabolism was monitored by indirect calorimetry and glucose kinetics was measured with 6,6-H_2 _glucose.

**Results:**

In both trials, LPS increased energy expenditure (p = 0.011), lipid oxidation (p<0.0001), and plasma lactate concentration (p = 0.016). In trial 1, lactate concentration in the muscle microdialysate was higher than in blood, indicating lactate production by muscles. This was, however, similar with and without LPS. In trial 2, calculated systemic lactate production increased after LPS (p = 0.031), while lactate clearance remained unchanged.

**Conclusions:**

LPS administration increases lactatemia by increasing lactate production rather than by decreasing lactate clearance. Muscle is, however, unlikely to be a major contributor to this increase in lactate production.

**Trial registration:**

ClinicalTrials.gov NCT01647997

## Introduction

Sepsis and inflammation elicit a whole set of neuroendocrine, metabolic, and systemic responses that belong to the organism's defense mechanisms [1,2]. However, when excessive, these responses may also exert deleterious effects [3]. Hyperlactatemia represents one prominent component of the metabolic stress response but shows marked inter-individual variations [4-6]. In critically ill patients, hyperlactatemia has been shown to be related to both the severity of the underlying condition and in-hospital mortality [7].

The mechanisms underlying the development of hyperlactatemia in sepsis remain poorly understood: increased production or decreased clearance or a combination of both are possible [8]. A rise in plasma lactate concentrations may result from a decreased lactate clearance secondary to low tissue oxygen delivery [9,10] or from an increase in lactate production secondary to anaerobic metabolism in under-perfused tissues or to stimulation of aerobic glycolysis in response to inflammatory mediators [11,12]. The relative importance of these two processes, as well as the major tissues where lactate metabolism is altered by sepsis, remains incompletely understood.

The impact of inflammation on the different metabolic pathways can be investigated by using a reproducible model of acute inflammation, consisting of endotoxin administration (lipopolysaccharide = LPS) [13], which closely mimics some important aspects of sepsis and has the advantage of being short-lived. LPS elicits the release of inflammatory mediators such as tumor necrosis factor-alpha (TNF-α) and interleukins (ILs) [14-17] and reproduces most of the neuroendocrine and metabolic alterations.

The present study aimed at investigating the relationship between inflammation and lactate metabolism and comparing local muscle responses and systemic metabolism. The objectives were to investigate the effect of endotoxin on the muscle lactate production in a first set of studies (trial 1) and on whole-body endogenous lactate production and lactate clearance in a second set of studies (trial 2). Since lactate is a major gluconeogenic precursor, an additional aim of this study was to evaluate the effects of an increased lactate supply on glucose production and glycemic control during endotoxin-induced sepsis-like conditions.

## Materials and methods

### Subjects and protocol

**Figure 1:**
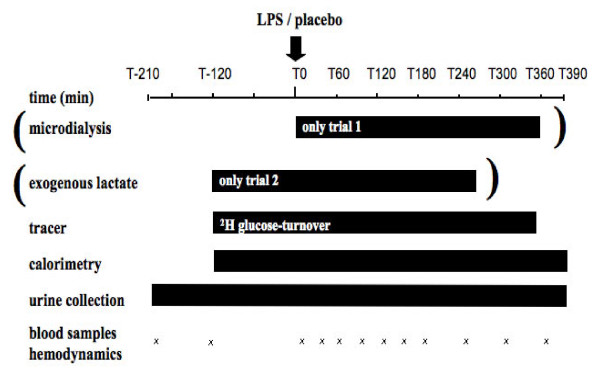
**Study design**. LPS, lipopolysaccharide.

The study was conducted with institutional ethics committee approval (Commission cantonale (VD) d'éthique de la recherche sur l'être humain) and individual signed informed consent. Fourteen healthy male volunteers were enrolled, and each subject took part in one of two consecutive parts of this study. The subjects were studied in a random order and on two occasions separated by at least one week: once with endotoxin administration (LPS day) and once with placebo (placebo day). Inclusion criteria were age of between 18 and 30 years and body mass index (BMI) of between 19.5 and 25. Exclusion criteria were family history of metabolic or endocrine disease, smoking, alcohol consumption, and current medication. The study design is summarized in Figure 1.

The subjects came to the metabolic laboratory at 7 a.m. after an overnight fast. Upon arrival, they were requested to void, and the urine was discarded. Thereafter, they remained supine quietly in a bed for the next 10 hours. An antecubital vein was cannulated for labeled tracer infusion. A dorsal hand vein of the opposite arm was cannulated to collected blood samples. This hand was placed in a thermostabilized box, which was heated at 50°C to achieve partial arterialization of venous blood.

After stabilization and tracer equilibration, endotoxin (LPS day: 2 ng/kg; US Pharmacopeial Convention, Rockville, MD, USA) or placebo (placebo day: NaCl 0.9%) was administrated as an intravenous bolus (T0).

Blood samples were collected at regular intervals up to 390 minutes later (T390) to determine glucose, triglycerides, free fatty acids (FFAs), insulin, glucagon, lactate, cortisol, adrenocorticotropine (ACTH), norepinephrine, epinephrine, TNF-α, IL-6, and C-reactive protein (CRP) concentrations and to determine plasma 6,6 ^2^H_2 _glucose isotopic enrichment. Blood samples were collected into heparinized tubes and centrifuged to separate plasma. Plasma was stored at -20°C (-80°C for catecholamines) until analysis.

Oxygen consumption and CO_2 _production measured by indirect calorimetry (by means of open-circuit indirect calorimetry as described previously [18]) were used to determine the energy expenditure [19]. Urine was sampled from spontaneous voiding throughout the study to assess the urinary urea nitrogen excretion. Heart rate, rectal temperature (Hellige; Servomed, Cairo, Egypt), arterial pressure, cardiac output by bioimpedance (NCCOM3 cardiodynamic monitor; BoMed, Irvine, CA, USA), and oxygen saturation (Biox 3740 Pulse oximeter; Ohmeda, now part of GE Healthcare, Little Chalfont, Buckinghamshire, UK) were monitored throughout the experiments.

#### Glucose turnover (trials 1 and 2)

After blood sampling for determination of basal glucose isotope enrichment and basal lactate, a primed continuous infusion of 6,6 ^2^H_2 _glucose (Cambridge Isotope Laboratory, Cambridge, MA, USA; prime 3 mg/kg per minute, continuous 30 μg/kg per minute) was infused from T180 (180 minutes before intravenous endotoxin or placebo) throughout the study.

#### Microdialysis (trial 1)

One microdialysis probe (CMA; Carnegie Medicine, Stockholm, Sweden) was inserted into the muscle vastus lateralis of one thigh and was infused with a Ringer solution containing 1 mmol/L ^13^C_3 _lactate (Cambridge Isotope Laboratory) at a rate of 0.3 μL per minute throughout the experiment. The cutoff of the microdialysis membrane was 30 kDa. Microdialysates were collected every hour to evaluate interstitial muscle lactate concentrations. The dialysate was collected at 60-minute intervals for measurement of dialysate lactate and ^13^C lactate concentrations.

#### Lactate clearance (trial 2)

Changes in lactate clearance and production elicited by endotoxin were evaluated by means of a continuous exogenous lactate infusion, as previously described [20]. Lactate (10 μmol/kg per minute) was infused from T90 until T270. Systemic lactate kinetics (lactate clearance and endogenous production) were assessed by means of a modified method of Connor and colleagues [21].

### Analytical procedures

#### Substrate analysis

Plasma glucose concentrations were measured enzymatically by using a YSI 2300 Stat Plus (YSI Incorporated, Yellow Springs, OH, USA), and plasma FFA concentrations were measured with a colorimetric method and a kit from Wako Chemicals GmbH (Neuss, Germany). Triglyceride levels were analyzed enzymatically by using the PAP 150 test (bioMérieux, Marcy l'Etoile, France). The urine nitrogen excretion rate was determined by means of the micro-Kjeldahl method. Concentrations of ACTH were determined with a chemiluminescence assay kit (Nichols Institute Diagnostics, San Juan Capistrano, CA, USA). Concentrations of insulin (kit from Adaltis, Casalecchio di Reno, Italy), cortisol (kit from Diagnostic Products Corporation, Los Angeles, CA, USA), and glucagon (kit from Linco Research, St. Charles, MO, USA) were determined by radioimmunoassay. Plasma concentrations of bioactive IL-6 and TNF-α were determined by using radioimmunoassay. CRP was analyzed by turbidimetry on a Hitachi 917 (Roche, Basel, Switzerland) by using reactants produced by Dako Schweiz AG (Baar, Switzerland). Plasma catecholamines were determined by high-performance liquid chromatography with electrochemical detection [22]. Plasma 6,6 ^2^H_2 _glucose isotopic enrichments were determined on deproteinized samples partially purified over sequential cation-anion exchange resins (AG 1-X8 and AG 50W-X8; Bio-Rad Laboratories, Richmond, CA, USA). For glucose measurements, pentaacetyl glucose was analyzed with gas chromatography-mass spectrometry (Hewlett-Packard, Palo Alto, CA, USA) [23]. Dialysate lactate concentration was measured by using a CMA microdialysis analyzer 600. The ^13^C lactate enrichment was measurement by gas chromatography-mass spectrometer, as previously described [24].

#### Calculations

Glucose rates of appearance and utilization (trials 1 and 2) were calculated from 6,6 ^2^H_2 _glucose by using Steele's equations for non-steady state [25]. Substrate oxidation and energy expenditure were calculated from respiratory gas exchanges and urinary urea nitrogen excretion rates by using the equations of Livesey and colleagues [26] corrected for urinary bicarbonate excretion rate [27].

For microdialysis measurements (trial 1), tissue substrate production is reflected by a positive gradient of concentration between the dialysate and the systemic circulation. This gradient was calculated as the difference of dialysate concentrations and systemic plasma concentrations, and a near-complete substrate recovery at the low flow rate (0.3 μL per minute) used in these experiments was assumed. Interstitial lactate concentrations were calculated by dividing dialysate lactate concentration by the recovery of infused ^13^C lactate, as described [24,28].

Lactate clearance (trial 2) was calculated as the quotient of exogenous lactate infusion rate and the plasma lactate difference (Δ lactate) induced by lactate infusion. Delta lactate was calculated from basal concentration (mean from T-150 to T-120) and plateau concentrations (mean from T-30 to T0) due to the start of lactate infusion as well as from plateau (mean from T240 to T270) and the fall to final lactate levels (mean from T360 to T390) due to the stop of lactate infusion. This procedure assumes that lactate concentrations do not vary significantly 5 hours after LPS administration. This has been documented in an earlier study performed in our lab [29].

#### Statistical analysis

Data were analyzed by using one-way or two-way analysis of variance (ANOVA) as appropriate. To determine whether lactate perfusion has an influence on the inflammatory response, two-way ANOVA was used to compare placebo groups of each trial. *Post hoc *analysis (Tukey test) was used in the presence of statistically significant changes. A *P *value of less than 0.05 was considered significant. The statistical package was JMP 8.0 (SAS Institute, Inc., Cary, NC, USA).

## Results

Characteristics of subjects were similar in the two trials (trial 1: n = 6; trial 2: n = 8): mean ages of 27.1 ± 2.4 and 26.7 ± 3.3 years, mean body weights of 74.8 ± 3.4 kg and 75.0 ± 6.9 kg, and mean BMIs of 24.0 ± 2.2 kg/m^2 ^and 23.9 ± 1.8 kg/m^2^, respectively.

### General and hemodynamic effects of lipopolysaccharide

**Figure 2:**
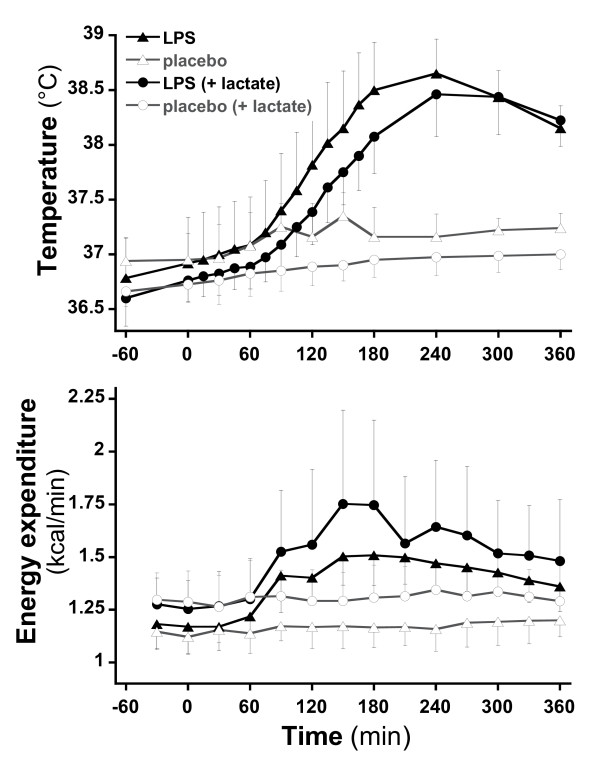
**Evolution of temperature and energy expenditure during the trial in four sessions**.

All subjects complained of headache, myalgia, and nausea. After LPS, significant increases in body temperature (mean peak at T240 of +1.7°C; *P *<0.0001) (Figure 2) and heart rate (mean peak of +38 beats per minute) (Figure 2) were observed, whereas mean blood pressure did not decrease significantly. Transcutaneous oxygen saturation remained normal in all subjects.

### Acute neuroendocrine and cytokine responses

**Figure 3:**
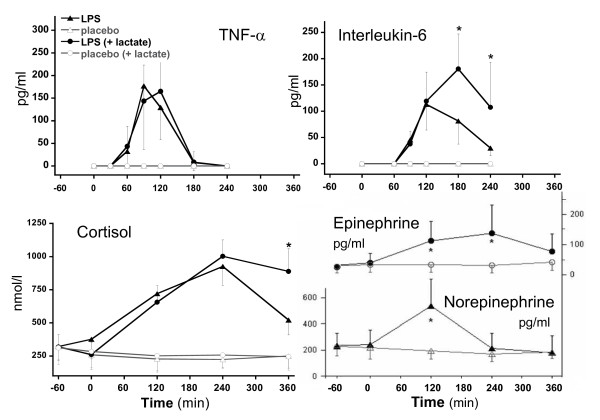
**Evolution over time of tumor necrosis factor-alpha (TNF-α), interleukin-6 cortisol, epinephrine, and norepinephrine after lipopolysaccharide (LPS) injection**. *P < 0.05.

After endotoxin administration, plasma cortisol (Figure 3) increased significantly (*P *<0.0001), peaking at T240. TNF-α also increased significantly (*P *<0.0001), peaking at T90, and was followed by a peak of IL-6 secretion between T120 and T150. Compared with trial 1, the increases in plasma IL-6 and cortisol were more sustained in trial 2 after exogenous lactate infusion. The stress hormones epinephrine and norepinephrine only were determined in trial 2 to have peaked at 120 minutes after LPS for norepinephrine and at 240 minutes after LPS for epinephrine.

### Metabolic effects

**Figure 4:**
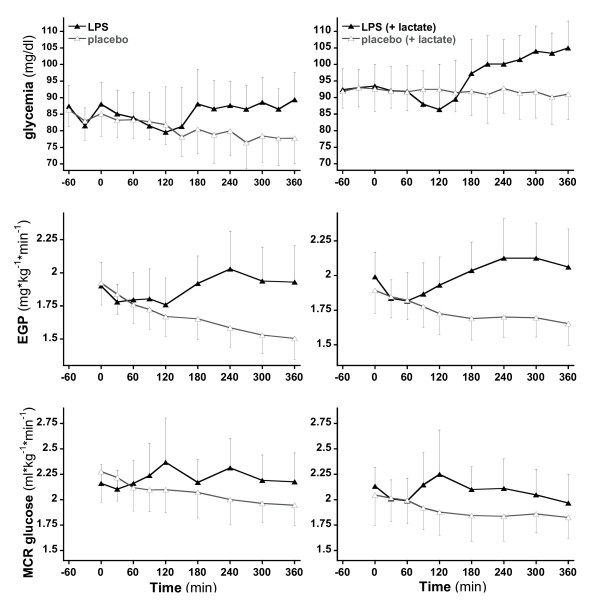
**Glycemia, endogenous glucose production (EGP), and metabolic glucose clearance (MCR) over time without (a) and with (b) exogenous lactate provision**.

Resting energy expenditure increased significantly in response to endotoxin (*P *= 0.011). Glycemia did not differ significantly at baseline. In both protocols, plasma glucose concentrations showed a biphasic response to endotoxin; an initial maximal decline occurred 2 hours after endotoxin administration, and a moderate rebound increase occurred between 3 and 6 hours after endotoxin (Figure 4). At T360, glycemia was still significantly higher in the endotoxin subjects of trial 2 in comparison with those of trial 1 (106 versus 91 mg/dL; *P *<0.0001). These changes were related to trends toward a reduced endogenous glucose production concomitant with a modest increase in metabolic glucose clearance early after endotoxin over the first hour after endotoxin, followed by an increase in glucose production, which was sustained throughout the remaining observation time. Endotoxin administration also elicited a 70% increase in plasma glucagon (from 64.6 to 107.4 ng/L at T120 in trial 1 and from 62.5 to 108.1 ng/L at T240 in trial 2) and enhanced the progressive increase in plasma FFA observed in placebo studies, as previously reported [30].

Plasma insulin concentrations were not affected by endotoxin. In trial 1, endotoxin significantly increased energy expenditure, and this increase was sustained over the 6-hour period of observation. A stimulation of lipid oxidation accounted for this increase in energy expenditure whereas glucose oxidation was unchanged.

### Lactate metabolism

**Figure 5:**
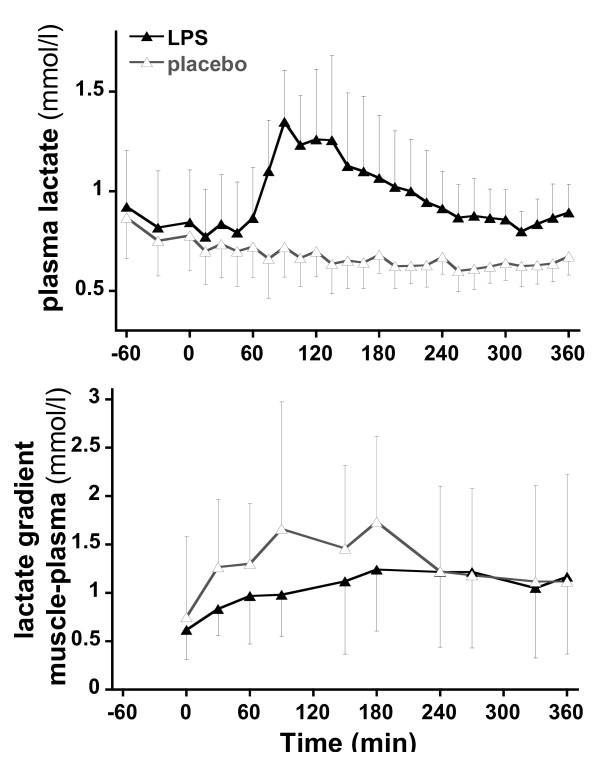
**Plasma lactate and difference between interstitial muscle and plasma lactate concentrations (lactate gradient of muscle to plasma) at various time points**.

In trial 1, LPS substantially increased the plasma lactate concentrations (Figure 5). Lactate concentrations peaked at T90 and declined progressively thereafter but remained higher than in the control experiment until the end of the observation period. Muscle interstitial lactate concentrations were systematically higher than plasma lactate concentrations, and their increase paralleled that of plasma lactate. The difference between interstitial lactate concentration and plasma lactate concentration (that is, lactate gradient from muscle towards plasma) (Figure 5) remained constant.

**Figure 6:**
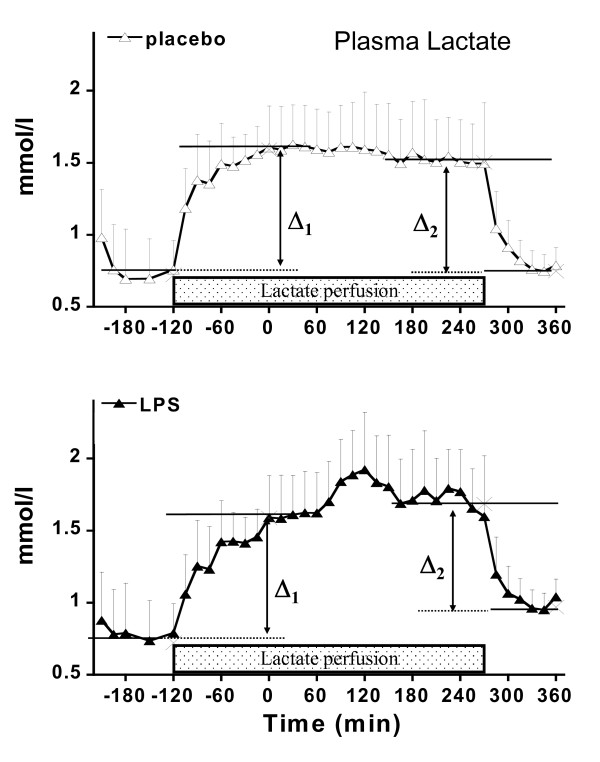
**Plasma lactate in trial 2 in control and lipopolysaccharide (LPS) phases**. Delta (Δ) values are the differences between basal lactate concentration and lactate measured during lactate infusion and were used to calculate whole-body lactate clearance. Since basal lactate was altered by LPS infusion, both the pre-LPS and post-LPS lactate concentrations were used as basal values to calculate (Δ1 and Δ2 values, respectively).

In trial 2, exogenous lactate infusion achieved a steady plasma lactate concentration during the 30-minute period preceding LPS administration and during the third hour following endotoxin administration (Figure 6). At the end of the exogenous infusion, plasma lactate declined rapidly in both LPS and placebo arms but stabilized at slightly higher values after endotoxin. Lactate production and clearance calculated during these periods are shown in Table 1. Lactate production was significantly increased after endotoxin, whereas lactate clearance was not altered.

**Table 1:** Lactate clearance and endogenous production in trial 2

Time point	Lactate clearance, mL/kg per minute	Endogenous lactate production, μmol/kg per minute
T0 (placebo)	12.4 ± 3.6	8.8 ± 2.6
T270 (placebo)	13.8 ± 2.8	10.2 ± 2.1
T0 (LPS)	12.1 ± 2.6	8.7 ± 3.0
T270 (LPS)	14.3 ± 2.2	13.9 ± 2.7^a^

Data presented as mean ± standard deviation. ^a^*P *= 0.031: Endogenous lactate production increased significantly in the lipopolysaccharide (LPS) group. T0, time point at 0 minutes; T270, time point at 270 minutes.

## Discussion

The present study corroborates earlier reports showing that an acute LPS challenge is a suitable model for the investigation of acute neuroendocrine and metabolic alterations elicited by bacterial infection and early sepsis [14-17,30]. Several key features of early sepsis - that is, acute transient increases in body temperature and heart rate, increases in plasma cytokines and stress hormone concentrations, a hypermetabolic state, and alterations of glucose homeostasis - were reproduced.

Administration of endotoxin increased total energy expenditure by about 35%. This increase was totally accounted for by a stimulation of lipid oxidation, whereas carbohydrate oxidation was not altered. This conclusion rests on the observation that, owing to fasting, net carbohydrate oxidation decreased continuously throughout the experiment but was similar in endotoxin and control experiments. Stimulation of lipolysis, presumably in adipose tissue [31], was documented by an accentuated increase in plasma FFA in comparison with control subjects and was most likely operative in fueling lipid oxidation.

Although endotoxin did not increase net glucose oxidation, it exerted significant effects on glucose homeostasis. In control subjects, a progressive decline in glucose production and glycemia was observed as a result of fasting. In contrast, administration of endotoxin tended to accentuate this drop in glucose production during the first hour but then consistently and continuously increased glucose production. This effect of endotoxin on glucose production is consistent with previous reports in humans and animals in response to administration of endotoxin and TNF-α and accounts for endotoxin-induced hyperglycemia since the metabolic clearance of glucose was essentially unaffected. Several studies have reported that bacterial endotoxin or administration of inflammatory cytokines induces a transient decrease in plasma glucose and an increase in glucose production and glucose utilization in fasted organisms [17]. Our present observations corroborate and extend these observations by showing that the increase in glucose utilization corresponds essentially to a stimulation of non-oxidative glucose disposal.

As a result of endotoxin administration, plasma lactate concentration increased consistently, peaked after 90 minutes, and progressively decreased thereafter. Plasma lactate, however, remained elevated throughout the 390-minutes observation period. Lactate concentrations in muscle interstitial fluid (trial 1) were significantly higher than plasma concentration throughout the experiment. This, however, was observed both in control experiments and after endotoxin and was not enhanced by endotoxin. This constant positive muscle-to-plasma gradient of lactate suggests that muscle lactate production was not increased by LPS. However, two limitations to the microdialysis technique need to be addressed. First, since muscle simultaneously produces and uses lactate, we cannot discard the hypothesis that both processes were increased to the same extent and hence that muscle lactate turnover was increased. However, if this was the case, it would not increase the net lactate efflux from muscle and therefore it would not participate in whole-body lactate production as measured in the present experiments. Second, an increased muscle lactate production may have resulted in a constant muscle-to-plasma lactate gradient if muscle blood flow had increased in the same proportion. Although this was not measured in our experiments, several reports indicate that muscle blood flow is not increased and may even be decreased after LPS in humans [32] and rats [33] and in patients with sepsis [34]. If muscle blood flow is assumed not to have increased in our experiments, the absence of an increased interstitial muscle-arterial gradient after LPS indicates that skeletal muscle produces lactate in fasting conditions but that this was not stimulated by endotoxin.

In trial 2, lactate kinetic was assessed by monitoring plasma lactate concentrations during continuous infusion of exogenous lactate. Continuous infusion of lactate is a variant of the bolus lactate administration approach [35-37], which we have described in detail elsewhere [20], and rests on the assumption that endogenous lactate production is not significantly affected by exogenous lactate, an assumption that is supported by a previous isotopic study [38]. Calculations of lactate clearance and production with such an approach require that plasma lactate concentration not vary markedly over time, and hence such calculations were possible only in basal conditions and during the fourth hour after endotoxin administration. For the latter, however, lactate concentrations tended to decrease over time in the placebo experiment of trial 2, implying that some underestimation of lactate production and overestimation of lactate clearance cannot be excluded. The data obtained from exogenous lactate infusion provide calculation of basal lactate production that were similar in both control and endotoxin experiments and were comparable to values reported in the literature with bolus lactate administration [35,36]. During the fourth hour after endotoxin administration, lactate clearance was unchanged, indicating that increased lactate production was responsible for the development of hyperlactatemia.

It may have been hypothesized that the increase in glucose production observed after administration of endotoxin was secondary to an increased gluconeogenesis from lactate. This hypothesis is consistent with the fact that the increase in lactate production (that is, about 4 μmol/kg per minute) closely matched the increase in glucose production (that is, about 0.5 mg/kg/min, corresponding to about 3 μmol/kg per minute). Our results obtained during lactate infusion, however, strongly suggest that the increase in glucose production was not directly dependent on lactate supply, since exogenous lactate did not further increase glucose production. Similar reports, in which lactate infusion stimulates gluconeogenesis but fails to enhance overall glucose production, have been reported in both healthy subjects [38] and critically ill patients [39]. This apparently surprising observation can be explained by an autoregulation of glucose production. According to this concept, glycemia per se regulates hepatic glucose output whereas acute alterations of gluconeogenesis lead merely to mirror changes in glycogenolysis [40]. Our present observation, by showing that endogenous glucose production is increased even though plasma glucose is higher (a factor that would normally inhibit hepatic glucose output), clearly indicates alterations of hepatic glucose metabolism. However, failure of exogenous lactate to further increase glucose production and glycemia indicates that hepatic glucose production is still efficiently autoregulated after endotoxin. This indicates (a) that increased gluconeogenic precursor availability is not the major factor responsible for an increase in glucose output and (b) that endotoxin administration increases fasting glycemia by resetting autoregulation of hepatic glucose production toward a higher glycemic level.

It must be emphasized, though, that our subjects had been fasted overnight before the test, and this affects glucose metabolism [41] but also the response to LPS, as in the trial by Fong and colleagues [42]. Depending on whether the clinician uses early enteral or parenteral nutrition, the flux of lactate may be affected differently in critically ill patients, and therefore our results in healthy subjects may not apply directly.

## Conclusions

These observations indicate that, in healthy humans, LPS administration leads to consistent increases in plasma lactate concentrations secondary to a stimulation of lactate production. However, the microdialysis data suggest that - provided that there was no increase in muscle blood flow - skeletal muscle was not a major contributor to LPS-induced lactate production. Further studies will be required to further evaluate which tissues are involved in LPS-induced lactate production, but immune cells and the reticulo-endothelial systems are obvious candidates as they are prime targets of cytokines secreted in response to LPS. Endotoxin administration increased fasting glucose concentration and endogenous glucose production, but this effect does not appear to be directly related to a stimulation of glucose production by enhanced lactate availability.

## Key messages

• Endotoxin administration leads to increased plasma lactate concentrations.

• Resting energy expenditure increased significantly in response to endotoxin.

• This increase is caused by a stimulation of glucose and lactate production.

• This increased lactate production does not occur in the muscle.

## Abbreviations

ACTH: adrenocorticotropine; ANOVA: analysis of variance; BMI: body mass index; CRP: C-reactive protein; FFA: free fatty acid; IL-6: interleukin 6; LPS: lipopolysaccharide; TNF-α: tumor necrosis factor-alpha.

## Competing interests

The authors declare that they have no competing interests.

## Authors' contributions

RLC, LT, J-PR, and MMB helped to conceive the study and draft and revise the manuscript. M-CC helped to conceive the study, conduct the trial, and acquire and analyze the data. AM helped to conduct the trial, acquire and analyze the data. BM helped to conduct the trial, acquire and analyze the data, and draft and revise the manuscript. All authors read and approved the final manuscript.
